# Ecosynthesis and Optimization of Nano rGO/Ag-Based Electrode Materials for Superior Supercapacitor Coin Cell Devices

**DOI:** 10.3390/ijms26199578

**Published:** 2025-10-01

**Authors:** Belen Orellana, Leonardo Vivas, Carolina Manquian, Tania P. Brito, Dinesh P. Singh

**Affiliations:** 1 Physics Department, Faculty of Science, University of Santiago of Chile (USACH), Avenida Victor Jara 3493, Estacion Central, Santiago 9170124, Chile; belen.orellana@usach.cl (B.O.); carolina.manquian@ce.ucn.cl (C.M.); tania.brito@usach.cl (T.P.B.); 2 Department of Electrical Engineering, Universidad Técnica Federico Santa María, Santiago 8940897, Chile; leonardo.vivas@usm.cl; 3 Departamento de Física, Facultad de Ciencias, Universidad Católica del Norte, Av. Angamos 0610, Antofagasta 1270709, Chile; 4 Centro Lithium I+D+i, Universidad Católica del Norte, Avenida Angamos 0610, Antofagasta 1270709, Chile

**Keywords:** ecosynthesis, graphene, rGO, Nano rGO/Ag, supercapacitor, coin cell device

## Abstract

In the shift toward sustainable energy, there is a strong demand for efficient and durable energy storage solutions. Supercapacitors, in particular, are a promising technology, but they require high-performance materials that can be produced using simple, eco-friendly methods. This has led researchers to investigate new materials and composites that can deliver high energy and power densities, along with long-term stability. Herein, we report a green synthesis approach to create a composite material consisting of reduced graphene oxide and silver nanoparticles (rGO/Ag). The method uses ascorbic acid, a natural compound found in fruits and vegetables, as a non-toxic agent to simultaneously reduce graphene oxide and silver nitrate. To enhance electrochemical performance, the incorporation of silver nanoparticles into the rGO structures is optimized. In this study, different molar concentrations of silver nitrate (1.0, 0.10, and 0.01 M) are used to control silver nanoparticle loading during the synthesis and reduction process. A correlation between silver concentration, defect density in rGO, and the resulting capacitive behavior was assessed by systematically varying the silver molarity. The synthesized materials exhibited excellent performance as supercapacitor electrodes in a three-electrode configuration, with the rGO/Ag 1.0 M composite showing the best performance, reaching a maximum specific capacitance of 392 Fg^−1^ at 5 mVs^−1^. Furthermore, the performance of this optimized electrode material was investigated in a two-electrode configuration as a coin cell device, which demonstrates a maximum areal-specific capacitance of 22.63 mFcm^−2^ and a gravimetric capacitance of 19.00 Fg^−1^, which is within the range of commercially viable devices and a significant enhancement, outperforming low-level graphene-based devices.

## 1. Introduction

Global warming has driven researchers worldwide to seek new sources of clean energy. While renewable energy offers multiple advantages, its availability can be limited by climatic and production factors, such as the intermittency of sunlight and wind, as well as energy conversion efficiency [1]. In this context, supercapacitors, particularly electrochemical double-layer capacitors (EDLs), have garnered increasing scientific interest due to their outstanding ability to deliver energy quickly and efficiently, in addition to offering higher cycle stability [2]. The operation of these energy storage devices is based on the separation of charge species, specifically through a non-faradaic charge–discharge process, enabling them to store electrical energy in the form of electrostatic charges [3]. Although their energy density is lower than that of commercially available batteries, their power density is substantially higher, making them an ideal choice for applications requiring instantaneous energy bursts, such as electric vehicles, cranes, regenerative braking in heavy vehicles, renewable energy systems, portable electronic devices, etc. [4,5].

Advanced carbon-based materials, such as graphene, activated carbon, or carbon nanotubes, have attracted significant attention for use as electrode materials due to their high performance [6]. Graphene is a two-dimensional structure consisting of a single layer of carbon atoms arranged in a hexagonal pattern [7]. An ideal graphene structure without defects (even the edge, which is already a defect, is not considered), containing only aromatic rings, has a surface area of approximately 2630 m^2^g^−1^, high electrical conductivity, and mechanical strength, which grant it superior electrochemical properties [8]. These characteristics make graphene an ideal candidate for energy storage applications, where efficiency and stability are essential. Additionally, graphene facilitates rapid ion diffusion, enhancing the charge and discharge rate of supercapacitors [9]. Another widely studied graphene-based material is graphene oxide (GO), obtained by introducing oxygen-containing groups into the hexagonal carbon lattice. Subsequent reduction of GO yields reduced graphene oxide (rGO), where some residual oxygen functional groups remain on the surface depending on the capacity of reducing agents [10]. Their presence reduces the electrical conductivity compared to pristine graphene but at the same time increases the number of active sites for electrochemical reactions, thereby enhancing the charge storage capability [11].

The incorporation of silver nanoparticles (AgNPs) not only enhances the electrical conductivity of the composite material but also increases the charge density and electrode stability [12]. These nanoparticles serve as additional charge accumulation sites, leading to a higher energy storage capacity and allowing the supercapacitor to store more energy within a given volume [13]. Functionalizing graphene with AgNPs is an effective strategy to further improve its electrochemical properties. Several studies have demonstrated that the integration of rGO and AgNPs into supercapacitors can enhance both capacitance and conductivity. Huang et al. reported that combining rGO with AgNPs improves energy storage capacity compared to bare rGO, showing a remarkable increase in specific capacitance and energy density [14]. These findings suggest that the synergy between graphene and AgNPs could play a key role in the development of high-performance supercapacitors.

The synthesis of AgNPs can be achieved through the chemical reduction of silver nitrate (AgNO_3_), a process that can be optimized by adjusting effective parameters such as reactant concentration, reaction time, pH, and temperature. Modifying these effective parameters influences the size and distribution of the nanoparticles, as well as their electrical properties, which directly affect the material’s conductivity and energy storage capacity [15,16].

Despite advancements in the design of materials for these devices, previous studies have not systematically evaluated the impact of different silver concentrations in composites based on rGO. Existing studies, such as the study by Kabiri et al., have typically reported the incorporation of AgNPs into rGO frameworks, but the influence of varying silver nitrate molarity on the structural and electrochemical properties of the resulting composites remains largely unexplored [17]. Furthermore, conventional synthesis protocols commonly involve separate reduction steps for GO and silver nitrate, often employing toxic or hazardous agents such as hydrazine or sodium borohydride. These methods raise concerns related to safety, environmental impact, and scalability [18].

In this work, a one-step green synthesis route was employed using ascorbic acid as a simultaneous reducing agent for both GO and AgNO_3_. This approach eliminates the need for harmful chemicals and simplifies the overall synthesis process. To investigate the effect of silver content on electrochemical performance, the molar concentration of AgNO_3_ was systematically varied (1.0, 0.10, and 0.01 M). These concentrations were selected to represent high, intermediate, and low silver loadings, respectively, covering a broad range that enables the evaluation of both extremes and the transition region. This approach enabled precise control over the incorporation of AgNPs and the defect density within the rGO grid, allowing for a systematic assessment of their combined effect on electrochemical behavior.

The synthesized materials were characterized structurally/microstructurally using techniques such as powder X-ray diffraction (pXRD), scanning electron microscopy (SEM), Raman spectroscopy, and Fourier Transform Infrared Spectroscopy (FT-IR) to confirm the incorporation of nanoparticles onto the surface of rGO. The electrochemical characterizations of the different composites were performed using cyclic voltammetry (CV), electrochemical impedance spectroscopy (EIS), and galvanostatic charge–discharge (GCD) tests in a three-electrode configuration in an electrolyte of 2 M KOH alkaline medium. Subsequently, an electrochemical characterization was carried out in a two-electrode configuration using the three composites. The same parameters as in the three-electrode configuration were studied, with the result showing that as the molar concentration of silver increases, the specific capacitance of the device also increases [19,20].

## 2. Results and Discussion

### 2.1. Structural and Spectral Characterization

FTIR, XRD, and Raman analyses were performed on pure rGO, and the corresponding results are provided in the supplementary material (supplementary information S1).

**Figure 1:**
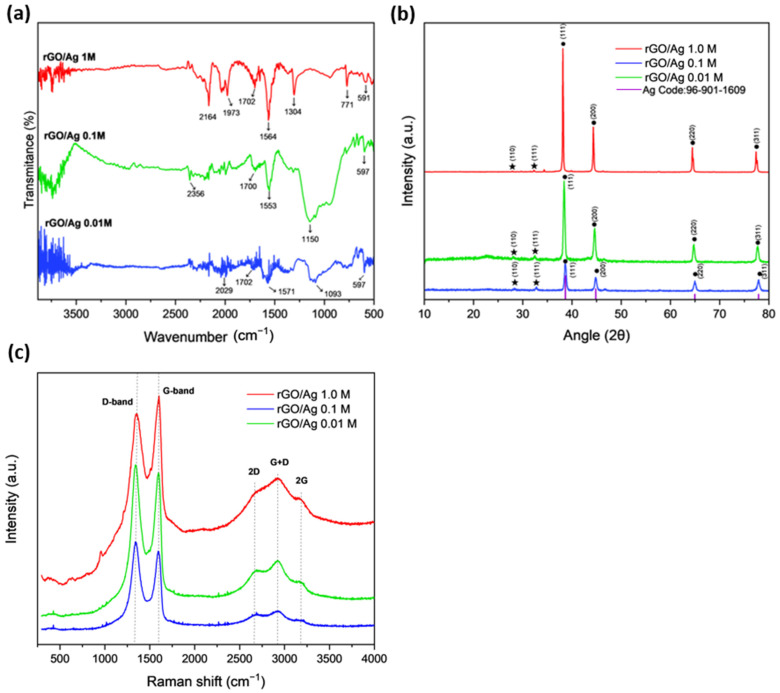
(**a**) FTIR spectra of rGO/Ag samples. (**b**) Powder X-ray diffraction patterns, where • denotes the characteristic peaks of metallic silver and ★ denotes the characteristic peaks of silver oxide. (**c**) Raman spectra of rGO/Ag samples.

Figure 1 presents the FTIR, XRD, and Raman results of the composites rGO/Ag 1.00 M, rGO/Ag 0.10 M, and rGO/Ag 0.01 M, where Figure 1a shows the FTIR spectrogram of the samples. In the rGO/Ag 1.0 M composites, a prominent peak at 2164 cm^−1^ is observed, associated with C≡C vibrations, indicating the presence of residual triple bonds [21]. These peak decreases in intensity in the samples with lower Ag concentration, suggesting a lower number of structural defects in these samples. In the region between 1702 and 1564 cm^−1^, peaks related to C=O (carbonyl) and C=C (aromatic graphene backbone) vibrations are observed [22]. The presence of peaks in 1702–1700 cm^−1^ reveals the existence of oxygen-containing functional groups present in the GO, indicating a partial reduction of the graphene oxide. In the rGO/Ag 1.0 M sample, the peak at 1702 cm^−1^ is more intense, indicating a higher amount of oxygenated functional groups. As the Ag concentration decreases, these peaks are attenuated, showing a progressive reduction in the number of oxygenated groups. This phenomenon is attributed to redox competition: Ag^+^ ions consume reducing agents during their nucleation, thereby limiting the complete reduction of epoxy and hydroxyl groups in GO [23]. Contrary to expectations, the C–O bands (1304–1150 cm^−1^) increase in intensity as the Ag concentration decreases. Recent studies have shown that AgNPs preferentially interact with residual C–O groups, masking their signal in samples with high metal content and thus explaining this apparent paradox [24,25]. Additionally, the peaks at 771 and 597 cm^−1^ correspond to out-of-plane C–H vibrations and metal–ligand interactions between rGO and the AgNP [26].

In Figure 1b, the XRD of different synthesized samples is shown, where • depicts the characteristic peaks of metallic silver and ★ shows the characteristic peaks of silver oxide. For the rGO/Ag 1.0 M sample, diffraction peaks are observed at the two Theta angles: 38.15, 44.35, 64.45, and 77.39°. For the rGO/Ag 0.1 M compound, the peaks are located at the two Theta angles: 38.30, 44.68, 64.74, and 77.75°, while for the rGO/Ag 0.01 M composite, the peaks are located at the two Theta angles: 38.64, 45.05, 65.36, and 78.18°. These peaks correspond to the crystallographic planes of metallic silver (111), (200), (220), and (311), respectively (JCSPDS no. 96-901-1609), which has the Fm-3m (225) space group, indicating a face-centered cubic (FCC) structure, where for all samples, the lattice parameter is a = 1.232 Å. The diffraction peaks of the samples show a shift toward larger angles, attributed to changes in the silver concentration, which directly affects the interplanar distances in the crystal structure. On the other hand, for all three samples, some very less intense characteristic peaks of Ag_2_O are also observed. For rGO/Ag 1.0 M, these are found at the 2 Theta angles: 27.83 and 32.27°, for rGO/Ag 1.0 M at the 2 Theta angles: 28.08 and 32.46°, and for rGO/Ag 0.01 M at the 2 Theta angles: 28.36 and 32.8°, corresponding to the (110) and (111) planes, respectively [27]. Additionally, in the rGO/Ag 1.0 M composite, a peak at 34.38° can be seen, indicating the formation of AgO. Furthermore, a peak at 22.80° is observed in the rGO/Ag 0.1 M sample and at 22.7° in the rGO/Ag 0.01 M sample, corresponding to rGO at a lower concentration, indicating that with the homogeneous formation of the composite, due to the higher Ag concentration in the 1.0 M sample, the graphene phase is not clearly distinguishable in the XRD analysis [28]. As shown in Figure 1b, most intense peaks correspond to either metallic silver or silver oxide. Due to high crystallinity and hence high intensity of the XRD peaks of Ag and Ag_2_O, the peaks corresponding to the rGO are suppressed and barely noticeable. Only a very-low-intensity, broad peak corresponding to the (002) plane of rGO appears around 23.8° in the samples rGO/Ag 0.1 M and 0.01 M, and it is completely negligible in the 1.0 M sample.

In Figure 1c, the Raman spectroscopy of the rGO composites in conjunction with AgNPs at different concentrations (1.0 M, 0.10 M, and 0.01 M) is presented. In the figure, three prominent peaks corresponding to the D, G, and 2D bands are observed, located around 1350, 1600, and 2700 cm^−1^, respectively, which confirm the formation of rGO. The D band is a vibrational mode that is activated by the presence of defects in the graphene lattice, while the G band is associated with the stretching of the C-C bonds in the sp^2^ hybridized carbon lattice [29,30]. The ratio between the intensities of the G and D bands, denoted as ID/IG, is an indicator of the degree of disorder in the material [31]. In the case of rGO/Ag 1.0 M sample, one can observe the ratio of the intensities of the G band (IG = 934.7) and D band (ID = 903.5), with IG/ID equal to 0.966, indicating the presence of a lower number of defects in the lattice due to the interactions with the AgNPs and then after reduction with a reducing agent. On the other hand, the intensities of the G and D bands in the case of the 0.1 M sample are IG = 661.0 and ID = 677.3, while for the 0.01 M sample, IG = 799.8 and ID = 812.7. Thus, the IG/ID ratios for the 0.1 and 0.01 M samples are 1.024 and 1.016, respectively. These values indicate a higher defect density compared to the 1.0 M sample4. This trend is consistent with the IR analysis, where a decrease in oxidized functional groups was observed for the 0.10 and 0.01 M samples, suggesting that GO underwent a more effective reduction, which in turn led to a higher density of structural defects compared to the 1.0 M sample, where the greater presence of oxygenated groups indicates that GO was not fully reduced.

The G+D peaks are activated due to the creation of defects and disorder on the surface caused by the chemical reduction of rGO [32]. The G+D peaks are located at 2921, 2926, and 2931 cm^−1^ for the rGO/Ag 1.0 M, rGO/Ag 0.1 M, and rGO/Ag 0.01 M samples, respectively. A shift toward lower frequencies is observed as the silver molarity decreases. This occurs, as previously mentioned, due to the increase in structural defects in the materials with lower amounts of AgNPs [33].

**Figure 2:**
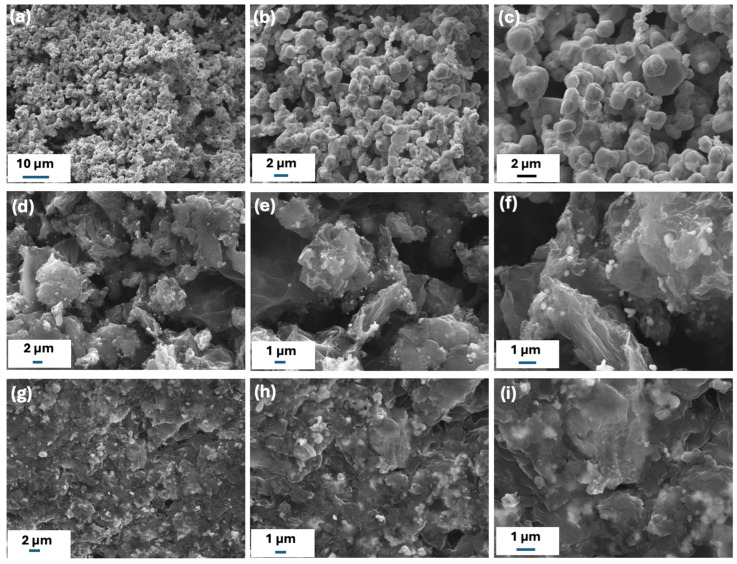
Low- and high-magnification SEM images of rGO/Ag samples synthesized at different molar concentrations of AgNO_3_ during synthesis. (**a**–**c**) rGO/Ag 1.0 M, (**d**–**f**) rGO/Ag 0.1 M, and (**g**–**i**) at rGO/Ag 0.01 M.

The microstructures of the synthesized samples were analyzed using scanning electron microscopy (SEM). Figure 2a–c presents the SEM images of rGO/Ag 1.0 M, where aggregated particles with an average size of 1.88 μm can be observed. Figure 2d–f displays the SEM images of rGO/Ag 0.10 M, where we can observe rGO flakes decorated with silver nanoparticles uniformly, with an average particle size of 320 nm. Figure 2g–i shows the SEM images of rGO/Ag 0.01 M, where we can see rGO sheets decorated with a smaller number of silver nanoparticles, with an average size of 625 nm, confirming the formation of silver nanoparticles.

The characterization study confirms the formation of graphene and AgNPs composites. However, it is also observed that silver concentration has a significant impact on the structure of these composites. At higher concentrations of silver nanoparticles, a decrease in structural defects is observed, accompanied by an increase in the number of oxygenated functional groups, due to the interactions between silver nanoparticles and the graphene matrix [34]. These interactions result in a partial rGO and promote the formation of secondary silver oxides, such as Ag_2_O and AgO. Additionally, SEM analysis reveals that silver concentration influences the morphology and particle size distribution of the composites. Lower Ag concentrations tend to yield smaller and more uniformly dispersed nanoparticles, whereas higher concentrations lead to particle aggregation and the formation of larger domains.

#### 2.1.1. Electrochemical Measurements in the Three-Electrode Configuration

The electrochemical performance of pure rGO was characterized by means of cyclic voltammetry (see Supplementary Information S2). As shown in Figure S2a, the voltammograms exhibit the characteristic behavior of electric double-layer capacitors (EDLCs). Figure S2b presents the specific capacitance of rGO as a function of scan rate. At the lowest scan rate of 5 mVs^−1^, a specific capacitance of 215 Fg^−1^ was obtained, while at the highest scan rate of 200 mVs^−1^, the capacitance decreased to 29.5 Fg^−1^. This reduction with increasing scan rate is typical of EDLCs, as at higher rates, ions have less time to diffuse into the inner pores of the material, thereby limiting the effective charge storage area [35]. Notably, 86% of the specific capacitance was lost when the scan rate increased to 200 mVs^−1^, indicating low stability under high-rate conditions.

**Figure 3:**
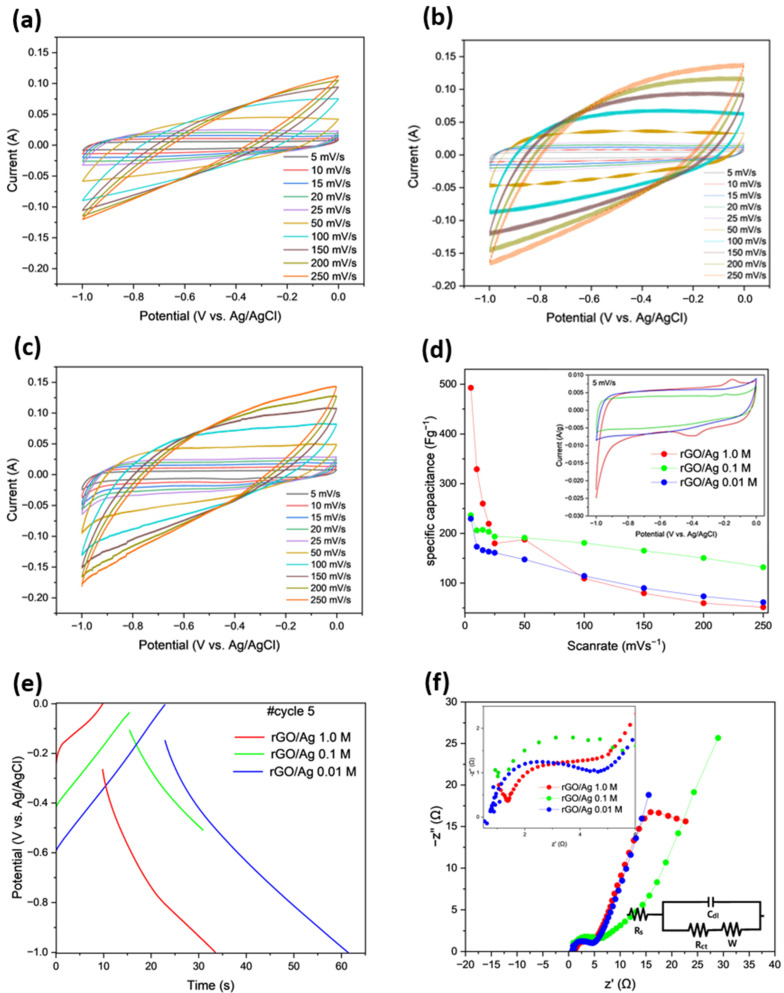
Electrochemical measurements of composites in a three-electrode configuration where (**a**) cyclic voltammetry of rGO/Ag 1.0 M, (**b**) rGO/Ag 0.1 M, and (**c**) rGO/Ag 0.01 M. (**d**) is the specific capacitance. (**e**) Charge and discharge curve of rGO/Ag 1.0 M, rGO/Ag 0.1 M, and rGO/Ag 0.01 M. (**f**) is the Nyquist plot.

The performance of the synthesized composites was evaluated through CV, GCD, and galvanostatic EIS. The electrochemical characteristics were investigated in a 2 M KOH alkaline medium. Figure 3a–c presents the CV curves performed in a potential window of [−1 to 0] V. In Figure 3a, it is observed that the rGO/Ag 1.0 M sample exhibits capacitive behavior at high sweep rates. In Figure 3b, the cyclic voltammograms of the rGO/Ag 0.1 M sample are shown, where peaks are observed at −1 V, along with a peak at −0.2 V. Figure 3c displays the rGO/Ag 0.01 M composite, which shows the characteristic behavior of double-layer supercapacitors (EDLCs) [36]. As shown in Figure 3d, at lower sweep rates, the CV curve displays pseudocapacitive behavior, with peaks at −0.2 and −0.4 V, indicating that the silver nanoparticles act as catalysts for fast surface redox reactions [37]. By using Equation (1), the specific capacitance of the samples was calculated. Figure 3d also presents the specific capacitance obtained for the rGO/Ag composites at different molar concentrations, with a minimum scanning rate of 5 mVs^−1^ and a maximum of 250 mVs^−1^. It can be observed that at a concentration of 1.0 M, the maximum capacitance is 492 Fg^−1^, while the minimum is 51 Fg^−1^; at a concentration of 0.1 M, the maximum capacitance is 236 Fg^−1^, and the minimum is 132 Fg^−1^; for a concentration of 0.01 M, the maximum and minimum capacitances are 229 and 61 Fg^−1^, respectively. Similarly, the capacitance of activated carbon (see Supplementary Information S3) decreases from 14 to 1 Fg^−1^ as the scanning rate increases from 5 to 250 mVs^−1^, demonstrating that activated carbon does not contribute significantly to the specific capacitance of the rGO/Ag composite electrodes. As observed, the capacitance of all four electrodes decreases as the scanning rate increases. This is because, at higher scan rates, diffusion limitations reduce ion accessibility to the internal regions of the electrode material, leaving the more accessible surface regions unaffected by diffusion constraints [38]. On the other hand, at lower scan rates, ions have enough time to diffuse deeper into the electrode material, allowing the use of internal sites that are inaccessible at high scan rates [39].

It can be observed from the results that the maximum specific capacitance was obtained with composite rGO/Ag 1.0 M. However, the electrode with the highest stability was observed for rGO/Ag 0.1 M, while varying the scan rate from low to high. It can be seen that there is only 44% loss in the specific capacitance when the scan rate increases, in comparison to 89% loss of specific capacitance in the case of the rGO/Ag 1.0 M composite. Instantly, the CV curves shown in the inset of Figure 3d look different than those of Figure 3a–c. However, at a lower scan rate of 5 mVs^−1^, it is the same as shown in the inset of Figure 3d. Due to the large number of curves at different scan rates, it is not clearly visible. Additionally, as diffusive pseudocapacitance predominates at lower scan rates, it rapidly decays when the scan rate increases due to the narrowing of the diffusion layer [40]. On the other hand, at lower silver concentrations, the distribution of these particles preserves the structure of the graphene, and the double-layer capacitive behavior predominates, which is less sensitive to changes in scan rates [41].

In Figure 3e, the galvanostatic CD tests of the different composites are shown. It can be observed that for rGO/Ag 0.1 M and rGO/Ag 0.01 M, the charge and discharge process exhibit a linear behavior, characteristic of ideal capacitive storage by double-layer capacitance. On the other hand, for rGO/Ag 1.0 M, the process shows pseudocapacitive behavior, which can be confirmed through cyclic voltammetry at 5 mVs^−1^ in Figure 3d. Additionally, it can be observed that the rGO/Ag 0.01 M sample has a longer charge and discharge time, taking a total of 60 s, while the rGO/Ag 0.1 M and rGO/Ag 1.0 M samples take around 35 s to complete the same process. By observing the potential difference that occurs between charge and discharge, it is noted that the rGO/Ag 1.0 M sample has a potential difference of 0.26 V, the rGO/Ag 0.1 M sample has a potential difference of 0.07 V, and the rGO/Ag 0.01 M sample has a potential difference of 0.14 V. From this, the internal resistance (R_esr_) can be determined as follows: for the rGO/Ag 1.0 M sample, R_esr_ = 13.38 Ω, for the rGO/Ag 0.1 M sample, R_esr_ = 5.14 Ω, and for the rGO/Ag 0.01 M sample, R_esr_ = 3.87 Ω. Therefore, with lower molar concentration, the internal resistance of the composites is low.

To analyze the EIS spectrum, an equivalent RC circuit was sought for the curves obtained. Figure 3f shows the EIS measurements of the three samples in a frequency range from 100 kHz to 0.10 Hz, along with the equivalent circuit of the obtained data. The total internal resistance of this circuit can be broken down into two main components: the series resistance (R_s_) and the charge transfer resistance (R_ct_). The series resistance R_s_ is due to the inherent resistance of the electrode’s active material, the resistance of the ionic electrolyte, and the contact resistance between the electrode and the current collector [42]. On the other hand, R_ct_, represented by the diameter of the semicircle in an EIS plot, is associated with the resistance due to Faradaic reactions occurring within the electrode’s porous structure [43]. Z_w_ represents the diffusive behavior related to ionic transport within the material. Finally, the vertical line results from the formation of the double-layer capacitor at the interface C_dl_ [44].

From Figure 3f and Table 1, the experimental values of R_s_, R_ct_, and C_dl_ can be obtained. For the rGO/Ag 1.0 M composite, the resistance R_s_ was 1.057 Ω, and the R_ct_ resistance was 3.187 Ω, with a double-layer capacitance C_dl_ of 49.940 µF, resulting in a leakage current of 313.70 µA. The rGO/Ag 0.1 M composite presented a slightly lower R_s_ of 0.930 Ω, but a higher R_ct_ of 3.734 Ω and a reduced capacitance C_dl_ of 42.620 μF, which corresponded to a leakage current of 267.80 µA. The rGO/Ag 0.01 M composite showed the lowest R_s_ of 0.626 Ω, yet the highest R_ct_ at 4.516 Ω, and the lowest C_dl_ among the three, at 35.240 µF. This sample also exhibited the lowest leakage current of 221.40 µA, indicating more restricted charge transport likely due to fewer active sites for redox reactions. This demonstrates the low resistivity of the composites and the electrolyte, highlighting their good electrochemical characteristics. As seen in the CD process, it is observed that as the molar concentration of Ag decreases, Rs decreases and R_ct_ increases. Figure 3f shows a trend line with an inclination close to 45° in the three materials studied, corresponding to the Warburg diffusive component, which describes the contribution of ion diffusion toward or away from the electrode surface [45]. In the case of rGO/Ag 1.0 M, the 45° slope evolves until it becomes parallel to the real axis, corresponding to a Warburg of finite length; this flattening indicates that, at low frequencies, the system acquires pure resistive behavior [46]. On the other hand, the rGO/Ag 0.1 M and rGO/Ag 0.01 M composites maintain an inclination of 45°, presenting semi-infinite diffusion.

#### 2.1.2. Electrochemical Measurements in Two-Electrode Configuration

Electrochemical measurements were performed in a two-electrode configuration on pure rGO and all three composites. The performance of the composites was evaluated using CV, galvanostatic CD, and EIS. The electrochemical characteristics were investigated in a 2 M KOH electrolyte.

The cyclic voltammetry curves of rGO exhibit the characteristic behavior of electric double-layer capacitors (EDLCs). The specific capacitance analysis revealed that at the lowest scan rate of 5 mVs^−1^, a specific capacitance of 9.8 Fg^−1^ was achieved, whereas at the highest scan rate of 250 mVs^−1^, the capacitance decreased to 2.5 Fg^−1^.

Cycling stability up to 2500 cycles showed that rGO retained 79% of its initial capacitance, demonstrating high electrochemical stability. The charge–discharge profiles were linear and symmetric, characteristic of ideal capacitive behavior. The total charge–discharge time was 19 s in the first cycle, slightly decreasing to 18 s by the 2500th, indicating minimal performance loss after prolonged use.

Electrochemical impedance spectroscopy (EIS) measurements were performed before and after cycling. Before CV, the R_s_ was 1.02 Ω, the R_ct_ was 7.80 Ω, and the C_dl_ was 5.45 µF. After CV, the values changed to R_s_ = 5.20 Ω, R_ct_ = 7.41 Ω, and C_dl_ = 7.42 µF. The slight increase in C_dl_ after cycling is related to improved ionic accessibility to the active surface after multiple cycles, while the variation in R_s_ could be attributed to minor changes in the electrolyte resistance or at the electrode–electrolyte interface (see supplementary information S4).

**Figure 4:**
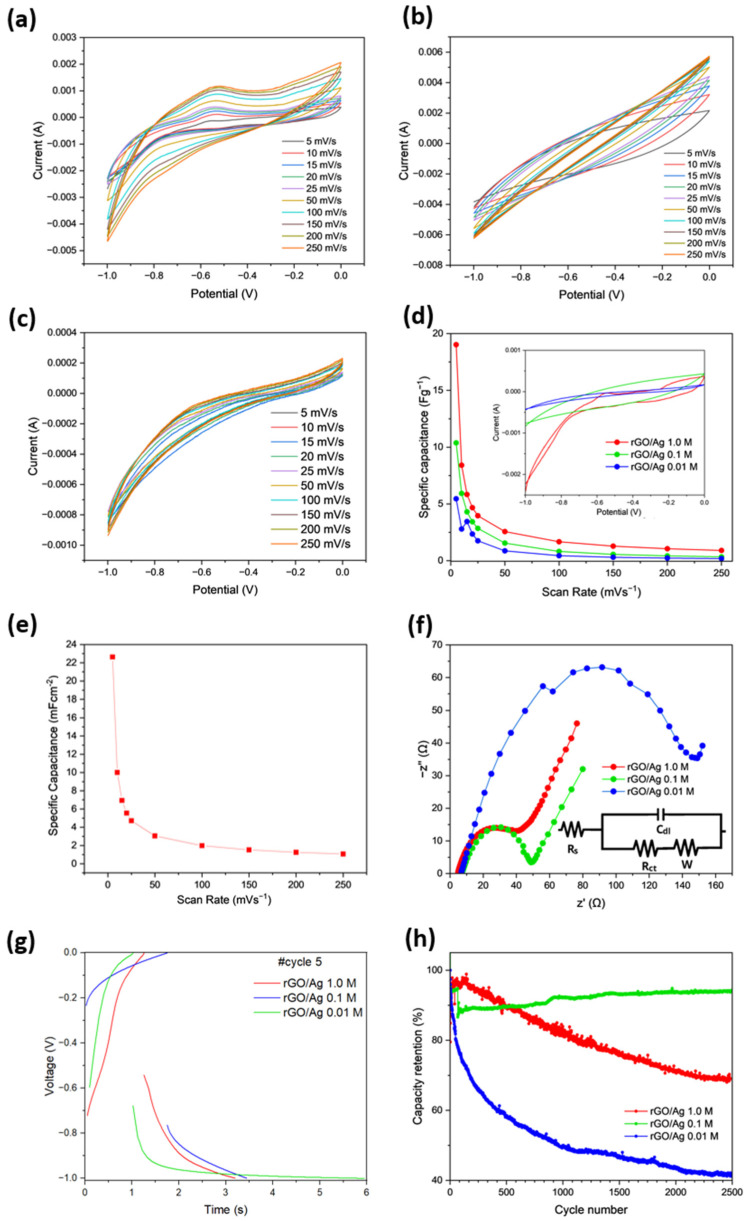
Electrochemical measurements of coin cell devices, where (**a**) cyclic voltammetry of rGO/Ag 1.0 M, (**b**) rGO/Ag 0.1 M, and (**c**) rGO/Ag 0.01 M, (**d**) measured specific capacitances, (**e**) the areal specific capacitance of rGO/Ag 1.0 M, and (**f**) the Nyquist plot. (**g**) Charge–discharge curves of the 5th cycle. (**h**) Cyclic stability and capacity retention.

Figure 4a–c presents the CV curves obtained in a potential window of [−1 to 0] V for rGO/Ag composites with varying silver concentrations. In Figure 4a, the CV curves of the supercapacitor are shown, exhibiting a pseudocapacitive behavior characterized by the presence of broad peaks associated with surface redox reactions and charge storage in the electric double layer. This behavior is consistent with what was previously observed in the three-electrode configuration for the rGO/Ag 1.0 M sample peaks at −0.54 V. In Figure 4b, the cyclic voltammograms of the rGO/Ag 0.1 M sample exhibit the characteristic behavior of double-layer supercapacitors (EDLC). Finally, Figure 4c shows the rGO/Ag 0.01 M composite, which also exhibits the typical behavior of EDLC. This behavior suggests that energy storage primarily occurs through ion adsorption–desorption processes on the material’s surface, which is typical for EDLCs [47]. The smaller enclosed area of the CV curves, compared to the other composites, indicates a reduced specific capacitance.

By using Equation (2), the specific capacitance of the samples is calculated. Figure 4d presents the specific capacitances for different molar concentrations of the composites, with a minimum scan rate of 5 mVs^−1^ and a maximum of 250 mVs^−1^. It can be observed that at a concentration of 1.0 M, the specific capacitance is 19 Fg^−1^, while the minimum is 0.9 Fg^−1^ at a scan rate of 250 mVs^−1^. For the rGO/Ag 0.1 M sample, the maximum capacitance was 10 Fg^−1^, while the minimum was 0.3 Fg^−1^ at the maximum scan rate. At a concentration of 0.01 M, the maximum specific capacitance was 5.4 Fg^−1^, while the minimum was 0.2 Fg^−1^. These values follow the trend of the measurements made in the three-electrode configuration, where the specific capacitance value is lower at a higher scan rate. It is also observed that the sample with the highest specific capacitance was that of 1.0 M.

In Figure 4e, the specific capacitance per unit area vs. scan rate of the rGO/Ag 1.0 M sample is shown, where the maximum value obtained was 22.63 mFcm^−2^ at 5 mVs^−1^, doubling the value of 10 mFcm^−2^ at the same scan rate reported by Bissett et al. for a graphene coin cell. This improvement is attributed to the incorporation of silver nanoparticles, which enhance electrical conductivity and facilitate ion diffusion [48].

To analyze the EIS spectrum, an equivalent RC circuit was plotted for the curves obtained. Figure 4f shows the EIS measurements of the three samples in a frequency range from 100 kHz to 0.10 Hz, along with the equivalent circuit of the data obtained. The total internal resistance of this circuit can be broken down into two main components: the series resistance (R_s_) and the charge transfer resistance R_ct_ (Inset Figure 4f). From Figure 4f and Table 2, the experimental values of R_s_, R_ct_, and C_dl_ can be obtained.

The rGO/Ag 1.0 M composite exhibits the lowest R_s_ of 4.900 Ω, an R_ct_ of 27.40 Ω, a high C_dl_ of 250.5 µF, and a leakage current of 36.50 µA, indicating efficient ion transport and favorable charge transfer kinetics. The rGO/Ag 0.1 M sample displays a higher R_s_ of 7.630 Ω and R_ct_ of 39.50 Ω, with a lower C_dl_ of 29.00 µF and a leakage current of 25.32 µA. These results suggest increased internal resistance and a reduction in interfacial capacitance.

Finally, the rGO/Ag 0.01 M sample presents an R_s_ of 7.060 Ω, the highest R_ct_ of 128.5 Ω, and the lowest leakage current of 7.78 µA. It has a C_dl_ value of 700.8 µF, indicating a substantial capacity for charge storage, despite the increased resistance. This demonstrates the low resistivity of the composites and the electrolyte, highlighting their good electrochemical characteristics. Figure 4f shows that all three composites exhibit semi-infinite Warburg diffusion. Comparing the results for rGO/Ag 1.0 M, diffusion is finite in the three-electrode configuration, whereas it remains semi-infinite in the two-electrode configuration. This difference is because, in the two-electrode cell, ionic diffusion does not intersect within the same frequency range because the diffusion path is longer and more symmetrical, shifting the transition point to lower frequencies outside the measured range [49]. Comparing the R_s_ results between the two and three-electrode configurations, the two-electrode setup shows a higher solution resistance. This is because the measured R_s_ in the two-electrode configuration includes not only the electrolyte resistance but also contributions from both electrodes and the current collectors. In contrast, in the three-electrode configuration, R_s_ primarily reflects the electrolyte resistance between the working and reference electrodes, with the reference electrode considered nearly ideal and contributing negligibly to the overall resistance. Consequently, R_s_ appears higher in the two-electrode configuration despite the shorter interelectrode distance [50]. Moreover, in a two-electrode configuration, electrode/electrolyte resistance is higher despite the shorter distance, due to poor contact and less availability of ions at the interface (as electrolyte KOH is soaked on filter paper only), in contrast with a large number of ions available in the liquid electrolyte in the case of a three-electrode configuration.

In Figure 4g, the galvanostatic CD test of the different composites is shown. It can be observed that for all samples, the charge and discharge process exhibit pseudocapacitive behavior, which can be confirmed through CV Figure 4a–c. Additionally, it can be observed that the rGO/Ag 0.01 M sample takes 6.0 s to complete the charge and discharge process, while the rGO/Ag 0.1 M and rGO/Ag 1.0 M samples take around 3.5 s to complete the same process. By observing the potential difference that occurs between charge and discharge, it is noted that the rGO/Ag 1.0 M sample has a potential difference of 0.54 V, the rGO/Ag 0.1 M sample has a potential difference of 0.67 V, and the rGO/Ag 0.01 M sample has a potential difference of 0.76 V. From this, the internal resistance (R_esr_) can be determined as follows: for the rGO/Ag 1.0 M sample, R_esr_ =186.21 Ω, for the rGO/Ag 0.1 M sample, R_esr_ = 101.52 Ω, and for the rGO/Ag 0.01 M sample, R_esr_ = 190 Ω.

Figure 4h illustrates the CV curve of the rGO/Ag composite-based device, which was tested through continuous CD cycles at a current density of 1 Ag^−1^ for 2500 cycles. The rGO/Ag 1.0 M material retained 80% of its initial capacitance after the last cycle, while the rGO/Ag 0.1 M sample retained 93% of its specific capacitance. The rGO/Ag 0.01 M sample, however, retained only 41% of its initial capacitance after 2500 cycles.

Comparing the results of the composites with pure rGO (see Supplementary Information S4) reveals some differences. Pure rGO, with a maximum capacitance of 9.8 Fg^−1^ at 5 mVs^−1^ and a minimum of 2.5 Fg^−1^ at 250 mVs^−1^, exhibited the typical behavior of an EDLC with good reversibility but limited energy storage capacity. After 2500 cycles, it retained 79% of its initial capacitance. In contrast, the rGO/Ag 1.0 M composite reached a maximum capacitance of 19 Fg^−1^, nearly doubling that of pure rGO, and showed a charge–discharge time of 3.5 s compared to 19 s for the material without silver, demonstrating a significant improvement in ion transport kinetics. Moreover, it retained 80% of its capacitance after 2500 cycles, offering higher energy storage capacity and efficiency.

The rGO/Ag 0.1 M composite, although with a lower capacitance of 10 Fg^−1^, exhibited the highest cycling stability, retaining 93% of its capacitance after 2500 cycles. Its low internal and charge transfer resistances enabled fast and efficient charge–discharge processes, making it the most durable material in the long term. On the other hand, the rGO/Ag 0.01 M composite, despite presenting an initial capacitance of 5.4 Fg^−1^ and a high C_dl_, showed the highest charge transfer resistance and the lowest cycling stability, indicating less favorable electrochemical performance.

**Figure 5:**
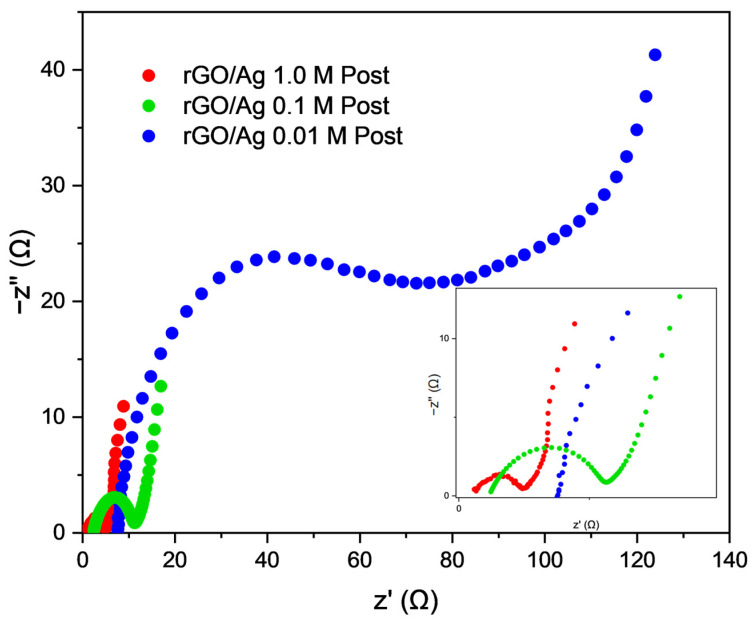
Post-galvanostatic impedance spectroscopy characterization of (Nyquist plots) of various rGO/Ag composites.

Post-characterization was performed using galvanostatic impedance spectroscopy after the charge and discharge process. Figure 5 shows the Nyquist plots for the rGO/Ag composites after cycling. The rGO/Ag 1.0 M composite exhibited an R_s_ of 1.354 Ω, an R_ct_ of 3.31 Ω, and a C_dl_ of 14.51 µF. For the rGO/Ag 0.1 M composite, the values were R_s_ = 2.5 Ω, R_ct_ = 8.28 Ω, and C_dl_ = 17.26 µF, whereas the rGO/Ag 0.01 M composite showed R_s_ = 8.02 Ω, R_ct_ = 45 Ω, and C_dl_ = 3000 µF. Compared with the initial values, the rGO/Ag 1.0 M and rGO/Ag 0.1 M composites exhibit only small increases in R_s_ and R_ct_, indicating that they maintain stable charge transfer kinetics and ionic conductivity after the voltammetry and cycling processes. This stability suggests that both composites preserve much of their electrochemical efficiency despite repeated charge–discharge cycles.

On the other hand, the rGO/Ag 0.01 M composite shows a considerable increase in charge transfer resistance, reflecting a more pronounced loss in charge transport efficiency across the electrode–electrolyte interface.

Despite these differences, all samples retain the Warburg diffusion component and exhibit semi-infinite diffusion behavior at low frequencies, confirming that ionic transport remains dominant even after the electrochemical characterization process.

From these results, it is confirmed that the incorporation of silver substantially improves the electrochemical performance of pure rGO. Among all of the composites, the rGO/Ag 1.0 M device exhibited the highest specific capacitance, delivering the best overall performance. However, considering the stability of the composites, the rGO/Ag 0.1 M sample displayed the best performance, as it had a longer cycle life. In contrast, the rGO/Ag 0.01 M composite exhibited both lower capacitance and poor cyclic stability. Table 3 shows a comparison of different rGO composites with AgNPs, electrochemically characterized. Compared to other approaches, the method used in our study stands out for its simplicity and sustainability. By employing a chemical reduction without dopants or high temperatures, it was possible to obtain composites with competitive specific capacitance, working in a wide potential window and using a lower electrolyte concentration.

The synthesis process reported in this work is also environmentally friendly, as it uses water as the main solvent, in contrast with other studies, wherein expensive solvents like 2,5-DABSA were utilized [54]. Furthermore, our samples were synthesized at room temperature, without high-temperature treatments, significantly reducing energy consumption compared to other reports that require temperatures up to 250–400 °C. Above all, simultaneous reduction of GO and silver nitrate by ascorbic acid not only reduced the requirement of two different reducing reagents for each reduction but also did so in an eco-friendly way. This one-step synthesis approach also provides a direct and cost-effective pathway to obtain high-performance rGO/Ag composites.

**Figure 6:**
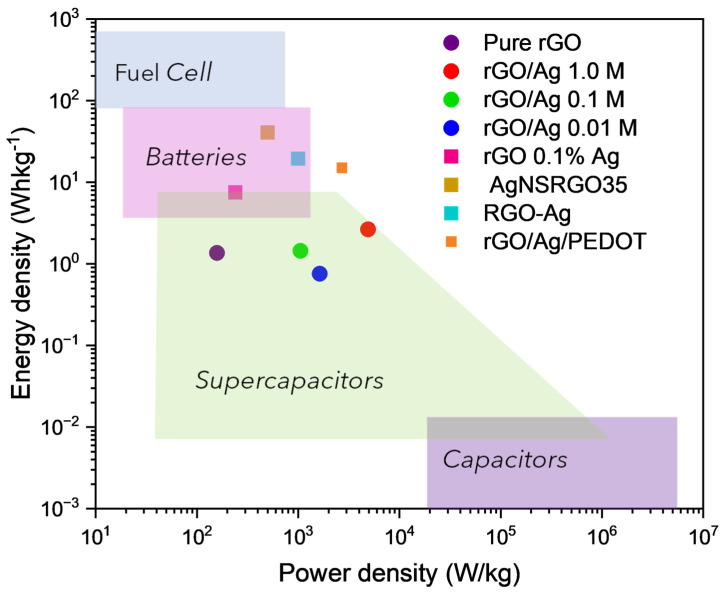
Ragone plot of the proposed supercapacitor devices, compared with previously reported systems based on graphene decorated with silver nanoparticles: rGO 0.1% Ag [51], AgNSRGO33 [52], RGO-Ag [53], and rGO/Ag/PEDOT [54].

Comparing these results with the data reported by Farbod et al., who describe an rGO electrode in a 2 M KOH electrolyte with an energy density of 250 Whkg^−1^ and a power density of 7.5 Wkg^−1^, it is evident that the rGO/Ag nanocomposites offer a significant improvement [55]. In particular, the high energy density observed in the nanocomposites suggests that the combination of rGO with AgNPs can optimize not only the electrochemical properties but also the energy storage capacity. Using the specific capacitance obtained from the CV curves (Figure 4b) at a scan rate of 5 mVs^−1^ and Equations (3) and (4), the Ragone plot for the assembled device can be obtained, which is shown in Figure 6.

This Figure illustrates the energy and power density ranges at which various devices operate, including the range for the devices under study. It highlights that the parameters of the investigated supercapacitors fall within the optimal operating range. Specifically, the energy densities are as follows: 2.64 Whkg^−1^ for rGO/Ag 1.0 M, 1.44 Whkg^−1^ for rGO/Ag 0.1 M, and 0.76 Whkg^−1^ for rGO/Ag 0.01 M. The power densities are 4903.6 Wkg^−1^ for rGO/Ag 1.0 M, 1054.3 Wkg^−1^ for rGO/Ag 0.1 M, and 1635.3 Wkg^−1^ for rGO/Ag 0.01 M. These values are comparable to those of devices fabricated using more complex methods, such as rGO/Ag/PEDOT-based nanocomposites, as reported by Ates et al., which exhibited an energy density of 15 Whkg^−1^ and a power density of 2700 Wkg^−1^ [56].

Pure rGO reaches 1.36 Whkg^−1^ and 258 Wkg^−1^, while the rGO/Ag composites significantly improve performance. The rGO/Ag 1.0 M composite achieves 2.64 Whkg^−1^ and 4903.6 Wkg^−1^, nearly doubling the energy density and increasing the power density by nineteen times. The rGO/Ag 0.1 M sample reaches 1.44 Whkg^−1^ and 1054.3 Wkg^−1^, with a moderate energy gain and a four times higher power density. In contrast, rGO/Ag 0.01 M delivers 0.76 Whkg^−1^ and 16,353 Wkg^−1^, reducing energy density but increasing power density, indicating a preference for fast charge–discharge capability rather than energy storage capacity.

The AgNPs incorporated into rGO composites enhance their electrochemical performance due to several synergistic effects. Silver is highly conductive, compensating for the inherent defects and incomplete reduction in rGO. The AgNPs act as conductive bridges, facilitating electron transport within the composite. Additionally, the presence of nanoparticles between rGO layers acts as spacers, preventing the restacking and aggregation of graphene sheets caused by van der Waals forces; this generates a higher surface area on the electrode and increases the accessible contact of the electrolyte with the electrode, thus increasing ionic absorption and therefore increasing capacitance [57].

The results show a nonlinear relationship between silver concentration and electrochemical performance in rGO/Ag composites. Although the rGO/Ag 1.0 M sample displayed the highest specific capacitance, this enhancement is primarily attributed to the increased pseudocapacitive contributions from silver redox reactions. However, this high silver content also led to a significant drop in capacitance retention and increased internal resistance, indicating reduced efficiency at high charge–discharge rates. In contrast, the rGO/Ag 0.1 M sample, while having a lower maximum capacitance, exhibited greater stability, lower internal resistance, and better capacitance retention.

Therefore, the optimization of AgNPs content is essential; higher concentrations enhance charge storage but at the cost of stability and efficiency, whereas intermediate concentrations strike a better balance between energy density and cyclic durability, positioning them as more practical candidates for real-world supercapacitor applications.

## 3. Materials and Methods

### 3.1. Materials

All chemicals and reagents used were of analytical grade and utilized as such. In the synthesis, such as 96% sulfuric acid (H_2_SO_4_) (Merck, Darmstadt, Germany), 85% orthophosphoric acid (H_3_PO_4_) ((Merck, Darmstadt, Germany), 99% potassium permanganate (KMnO_4_) (Merck, Darmstadt, Germany), 30% hydrogen peroxide (H_2_O_2_) (Sigma-Aldrich, St. Louis, MO, USA), graphite powder <20 μm (Sigma-Aldrich, St. Louis, MO, USA), anhydrous silver nitrate (AgNO_3_) (ChemiX), ascorbic acid, and the reagents for electrochemical analysis, such as polyvinylidene fluoride (PVDF) (Sigma-Aldrich, St. Louis, MO, USA 1-methyl-2-pyrrolidone (NMP) (Sigma-Aldrich, St. Louis, MO, USA), potassium hydroxide (KOH) (EMSURE^®^, Darmstadt, Germany), activated carbon (AC) (Supelco Analytical, St. Louis, MO, United States), and conductive silver paint (Spi Supplies) were purchased from various suppliers and used without further purification.

### 3.2. Synthesis Methods

#### 3.2.1. Synthesis of Graphene Oxide (GO)

GO was synthesized by using the well-established modified Hummers’ method [58]. In this method, in an ice bath, a solution of 22.5 mL of H_2_SO_4_ and 0.50 g of graphite powder was prepared. This mixture was stirred constantly for 30 min. Thereafter, 2.5 mL of H_3_PO_4_ was added, and stirring continued for 1 h. Subsequently, 3.0 g of KMnO_4_ was slowly added to the solution and stirred for 60 min. Once mixing was completed, the solution was removed from the ice bath, and 100 mL of distilled water was added, with stirring continued for 24 h at room temperature. Finally, 10 mL of H_2_O_2_ solution was added, resulting in a light brown colloidal solution corresponding to GO. This suspension was washed with distilled water several times to remove impurities, soluble by-products, and residual reagents.

#### 3.2.2. Synthesis of rGO/Ag Composites

**Figure 7:**
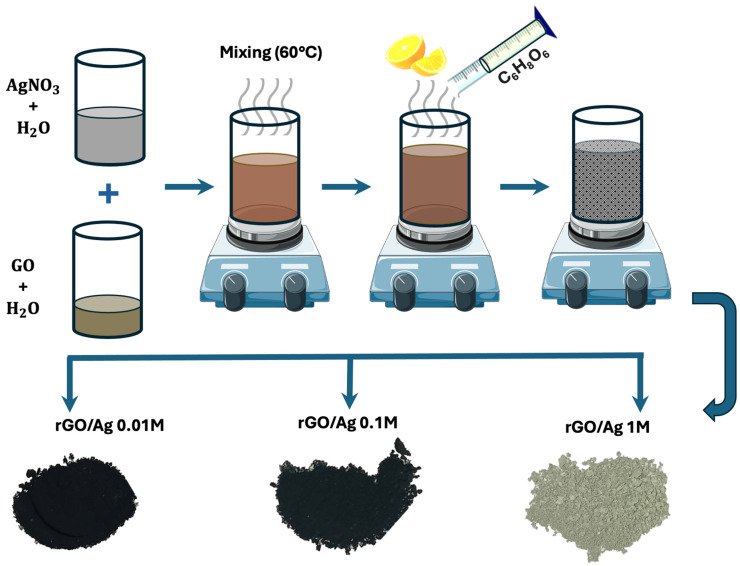
Experimental method for the synthesis of the rGO/Ag composite and optical images of the as-obtained product at different molar concentrations of AgNO_3_ (0.01 M, 0.1 M, and 1.0 M).

For the preparation of the composites, 20 mL of the as above obtained GO was diluted in 100 mL of distilled water and stirred for 30 min. Simultaneously, different molar concentrations of anhydrous AgNO_3_ (1.0, 0.10, and 0.01 M) were prepared in 200 mL of distilled water and stirred for 30 min on another, separate magnetic stirrer. Once both solutions were prepared, the AgNO_3_ solution was added to the GO solution (in situ), under constant magnetic stirring conditions for 30 min. Subsequently, the mixture was heated to 60 °C and maintained at this temperature for 30 min. Next, 100 mL of an aqueous solution of ascorbic acid with a molar concentration of 0.57 M (10 g) was slowly added (~5 mL per minute) and stirred for 1 h at 60 °C, as described in Figure 7. Finally, the mixture was cooled to room temperature, washed several times with distilled water, and then dried at room temperature and stored.

### 3.3. Structural, Microstructural, and Spectral Characterization

The structure of the synthesized materials was analyzed through the powder X-ray diffraction (pXRD) technique by using a Bruker D-8 Advance X-ray diffractometer (Bruker AXS, Karlsruhe, Germany) with CuKα radiation (1.5406 Å). Diffraction was measured at a 2-theta angle, ranging from 10° to 80°, with a step size of 0.02° s^−1^. Microstructural and elemental characterization was carried out by using a Zeiss Evo MA10 (Carl Zeiss Microscopy, Oberkochen, Germany) scanning electron microscope (SEM) operated at an accelerating voltage of 20 kV. For spectral characterization, Raman spectroscopy was used with an NRS-4500 Jasco spectrometer, equipped with a 532 nm laser, in a range of 450 to 4000 nm, and Fourier-transform infrared (FT-IR) spectroscopy was performed with a Bruker IFS66V (Billerica, MA, USA), in a wavelength range from 450 to 4000 nm.

### 3.4. Electrochemical Characterization

#### 3.4.1. Electrode Preparation

For the preparation of the working electrode, a weight ratio of 7:2:1 = active material–activated carbon–polyvinylidene fluoride (PVDF) was used, respectively. To prepare the mixture homogeneously, 1.5 mL of NMP was also added and stirred for 1 h. Subsequently, the mixture was heated to 60 °C to evaporate the solvent and transform the mixture into a paste. This paste was spread homogeneously on a nickel foam with an area of 1.0 cm^2^ and left to dry at room temperature for 24 h to allow the complete evaporation of the solvent. Finally, the nickel foam was connected to a copper wire with conductive silver paint. The loading mass of each fabricated electrode was approximately 20 mgcm^−2^.

#### 3.4.2. Standard Three-Electrode Configuration

**Figure 8:**
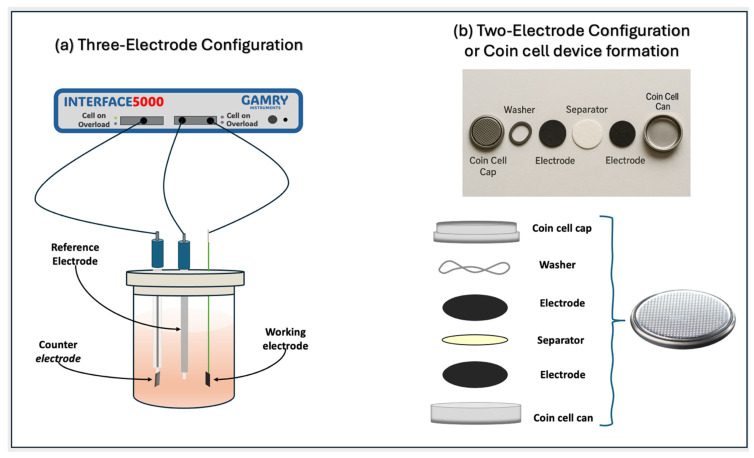
Electrochemical measurements in (**a**) the three-electrode configuration and (**b**) the symmetrical two-electrode configuration or coin cell supercapacitor device fabrication.

The electrochemical properties were studied using a three-electrode configuration, a working electrode, platinum (Pt) as a counter electrode, and Ag/AgCl as a reference electrode, in a 2.0 M KOH alkaline electrolyte solution, as shown in Figure 8a. All of the electrochemical measurements were performed by using the i5000E Gamry potentiostat, and the data were processed in Gamry Analysis software. The electrochemical characterizations were performed using cyclic voltammetry (CV), galvanostatic electrochemical impedance spectroscopy (EIS), and charge–discharge (CD) in a potential window of [−1 to 0] V. Different sweep rates were used (5.0, 10, 15, 20, 25, 50, 100, 150, 200, and 250 mVs^−1^) for CV tests. Electrochemical impedance spectroscopy (EIS) measurements were performed in the frequency range from 100 kHz to 0.10 Hz. The charge–discharge curves were analyzed in a potential window of [−1 to 0] V using a current density of 1.0 Ag^−1^. Specific capacitance was calculated from the CV curves using the following equation:

(1) $$C_{s}[Fg^{-1}]=\frac{\int idV}{\Delta V\cdot \nu \cdot m_{ef}}$$

where ∫idV corresponds to the voltammetry integral between the upper and lower limits of the potential window ΔV (V).  ν represents the sweep rate (mVs^−1^), and $m_{ef}$ is the effective mass of the electrode composites.

#### 3.4.3. Two-Electrode Configuration or Coin Cell Device Formation

Electrochemical tests were performed in a two-electrode set using a coin cell device. The working electrodes were prepared as described in the three-electrode configuration, using a stainless-steel substrate instead of nickel foam. The same mass was used for both symmetrical electrodes and dried at room temperature. The electrodes were separated by a commercial filter paper soaked in a 2 M KOH solution. This configuration was inserted between two caps and a spring and then pressed with a hydraulic pressure machine at a pressure of 1.33 MPa to obtain the coin cell, as shown in Figure 8b. The measurements were made using the Reference 3000 Gamry instrument, and the data were processed using the Gamry Analysis software version 7.8.3. The electrochemical characterization was performed using the same parameters and conditions as those used for the three-electrode configuration. Specific capacitance can be calculated from the CV curves using the following equation:

(2) $$C_{s}\left[Fg^{-1}\right]=\frac{\int idV}{2\cdot \Delta V\cdot \nu \cdot m_{ef}}$$

where ∫idV corresponds to the voltammetry integral between the upper and lower limits of the potential window, ΔV (V).  ν represents the sweep rate (mVs^−1^), and $m_{ef}$ is the effective mass of the electrode composites.

To evaluate the energy density and power density per unit mass, the following equations were used:

(3) $$E_{s}\left[Wh{kg}^{-1}\right]=\frac{1}{2}C_{s}V^{2}$$

where $C_{s}$ is the specific capacitance in farads per gram (Fg^−1^) and V is the applied voltage (V), and,

(4) $$P\left[W{kg}^{-1}\right]=\frac{E_{s}}{\Delta t}$$

where Δt is the discharge time in (h).

## 4. Conclusions

A highly efficient, low-cost, easy-to-fabricate, commercially viable, and eco-friendly green supercapacitor has been developed using rGO composites decorated with AgNPs. The coin cell supercapacitor device exhibits a maximum areal capacitance of 22.63 mFcm^−2^ and a specific capacitance of 19.00 Fg^−1^. The device retains 80% of its initial capacitance after 2500 cycles, demonstrating excellent stability and durability.

This green synthesis method employs ascorbic acid as a reducing agent, avoiding toxic chemicals such as hydrazine and ensuring an environmentally friendly process. The incorporation of AgNPs enhances conductivity. This scalable and simple synthesis approach can be extended to other metal–graphene composites (e.g., Cu or Au) for broader applications in energy storage.

The electrochemical performance of the device, characterized by cyclic CV, GCD, and EIS, revealed a low internal resistance (R*_S_* = 4.91 Ω) and a moderate charge transfer resistance (R*_ct_* = 33.02 Ω), indicating excellent ionic and electronic conductivity. These results position the rGO/Ag composite as a promising candidate for next-generation supercapacitors, with great potential for commercialization in portable electronic devices and renewable energy systems.

## Figures and Tables

**Table 1:** Experimental values obtained from the Nyquist plot.

Composite	R_s_ [Ω]	R_ct_ [Ω]	C_dl_ [µF]	Leakage Current [μA]
rGO/Ag 1.0 M	1.057	3.187	49.940	313.70
rGO/Ag 0.1 M	0.930	3.734	42.620	267.80
rGO/Ag 0.01 M	0.626	4.516	35.240	221.40

**Table 2:** Calculated experimental values from the Nyquist plot in the two-electrode configuration.

Composite	R_s_ [Ω]	R_ct_ [Ω]	C_dl_ [µF]	Leakage Current [μA]
rGO/Ag 1.0 M	4.900	27.40	250.5	36.50
rGO/Ag 0.1 M	7.630	39.50	29.00	25.32
rGO/Ag 0.01 M	7.060	128.5	700.8	7.78

**Table 3:** Various reports related to graphene- and silver-based materials for supercapacitors.

Materials	Cs	Electrolyte	Potential Window	Reference
rGO 0.01% Ag 0.1% Ag	209.32 Fg^−1^ 215.62 Fg^−1^ at 1 Ag^−1^	6 M KOH	[−1.0–0.0]	[51]
AgNSrGO35	923.3 Fg^−1^ at 1 Ag^−1^	6 M KOH	[−1.2–0.0]	[52]
rGO-Ag PANI rGO.Ag	296.7 Fg^−1^ at 5 mVs^−1^ 140 Fg^−1^ at 0.5 Ag^−1^ 385.4 Fg^−1^ at 5 mVs^−1^ 194.4 Fg^−1^ 0.5 Ag^−1^	6 M KOH	[0.0–1.0]	[53]
rGO/Ag 1.0 M	392 Fg^−1^ at 5 mVs^−1^ 19 Fg^−1^ coin cell at 5 mVs^−1^ At 1 Ag^−1^	2 M KOH	[−1.0–0.0]	This work
rGO/Ag 0.1 M	236 Fg^−1^ at 5 mVs^−1^ 10 Fg^−1^ coin cell at 5 mVs^−1^ At 1 Ag^−1^	2 M KOH	[−1.0–0.0]	This work
rGO/Ag 0.01 M	229 Fg^−1^ 5.4 Fg^−1^ coin cell at 5 mVs^−1^ At 1 Ag^−1^			

## Data Availability

The original contributions presented in this study are included in the article/Supplementary Materials. Further inquiries can be directed to the corresponding author.

## Supplementary Materials

The following supporting information can be downloaded at: https://www.mdpi.com/article/10.3390/ijms26199578/s1. References [10,21,22,26,32] are cited in the supplementary materials.
