# Supplementary material for: Ecosynthesis and Optimization of Nano rGO/Ag-Based Electrode Materials for Superior Supercapacitor Coin Cell Devices
Source: Int J Mol Sci. 2025 Oct 1;26(19):9578. doi: 10.3390/ijms26199578 (PMC12524916; doi:10.3390/ijms26199578)
Supplement: Supplementary file 1 [file ijms-26-09578-s001.zip › ijms-3848097-supplementary.pdf]

Supplementary Information

S1. Structural and Spectral Characterization of rGO

Figure S1 (a) shows the FTIR spectrum of rGO, where a peak at 2164 cm⁻¹ is attributed to C≡C vibrations, indicating the presence of residual triple bonds in the structure [10]. In the region 1753–1652 cm⁻¹, signals associated with C=O (carbonyl) and C=C (aromatic graphene backbone) vibrations appear [21]. The peaks around 1753 cm⁻¹ confirm the presence of oxygen-containing groups, consistent with evidence of incomplete reduction of GO. Likewise, the bands in the 1314–1136 cm⁻¹ range correspond to C–O groups, while the peaks at 1021 and 752 cm⁻¹ are related to out-of-plane C–H vibrations, characteristic of aromatic structures [22].

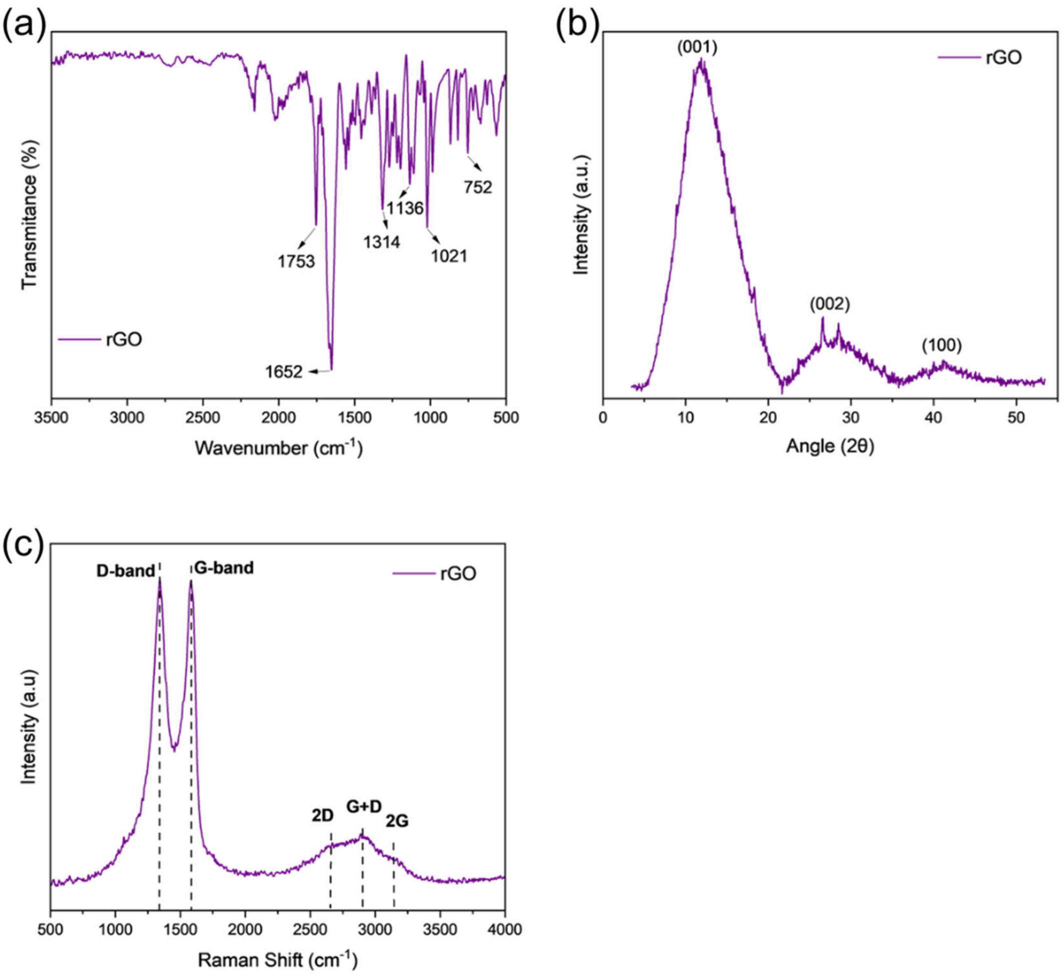

Figure S1. (**a**) FTIR spectra of rGO sample**.** (**b)** Powder X-ray diffraction pattern rGO sample. (**c**) Raman spectra of rGO sample.

Figure S1 (b) presents the X-ray diffraction (XRD) pattern of rGO, showing characteristic peaks at 11.67, 26.6, and 41.9°, corresponding to the (001) plane of GO and the (002) and (100) planes of rGO, respectively [26]. The simultaneous presence of these planes suggests

that the reduction of graphene oxide (GO) by ascorbic acid was partial, with some oxygenated regions remaining while others were reduced to form rGO.

Additionally, Figure S1 (c) presents the Raman analysis of rGO, revealing three prominent peaks corresponding to the D, G, and 2D bands, located at approximately 1350, 1600, and 2700 cm^{-1}, respectively. The intensity ratio ID/IG =0.99 suggests a moderate level of structural defects, consistent with a partial reduction process [32]. Overall, the characterization confirms the formation of partially reduced rGO.

S2. Electrochemical Measurements of rGO in Three-Electrode Configuration

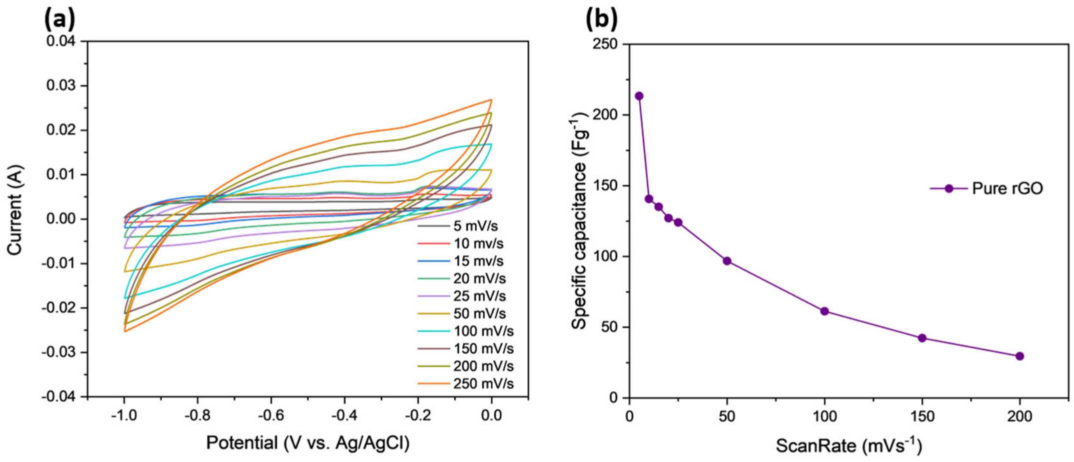

Figure S2. Electrochemical measurements of rGO in a three-electrode configuration where **(a)** Cyclic voltammetry **(b)** the specific capacitance.

S3. Specific Capacitance Activated carbon

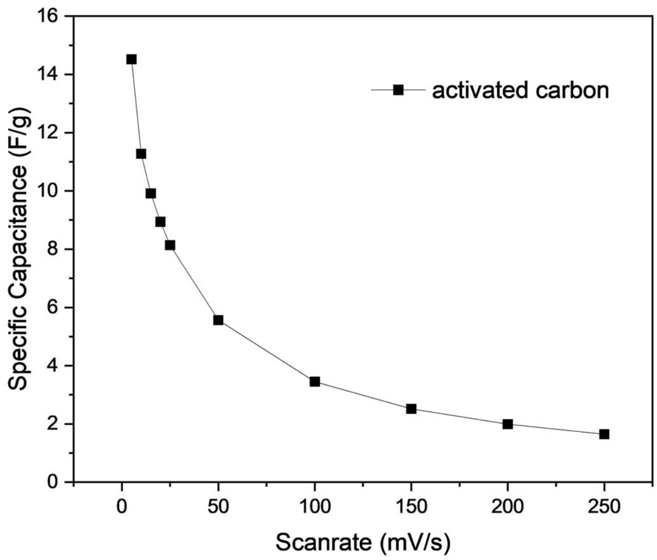

Figure S3. Specific capacitance of activated carbon at different scan rates.

S4. Electrochemical Measurements of rGO in Two-Electrode Configuration (Coin Cell)

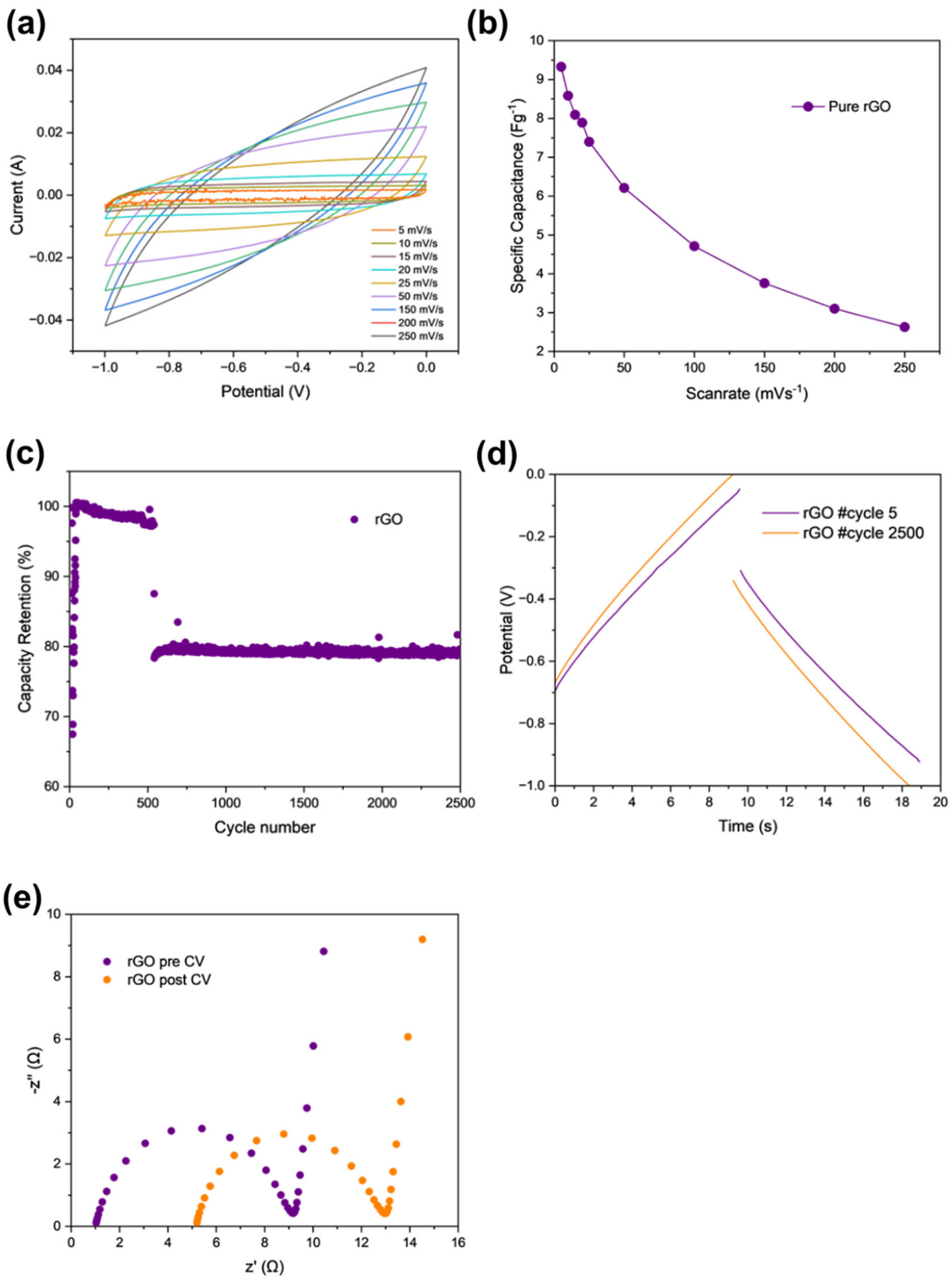

Figure S4. Electrochemical measurements of rGO in a two electrode configuration where **(a)** Cyclic voltammetry **(b)** the specific capacitance**.** **(c)** Capacity retention. (**d)** Charge and discharge curve at cycle 5 and cycle 2500, and **(f)** is the Nyquist plot before CV and after CV.

References:

[10] M. S. Amir Faiz, C. A. Che Azurahanim, S. A. Raba'ah, and M. Z. Ruzniza, "Low cost and green approach in the reduction of graphene oxide (GO) using palm oil leaves extract for potential in industrial applications," *Results Phys*, vol. 16, Mar. 2020, doi: 10.1016/j.rinp.2020.102954.

[21] M. Shaban, A. Mohamed, M. G. M. Kordy, H. AlMohamadi, M. F. Eissa, and H. Hamdy, "Design and Performance of CuNi-rGO and Ag-CuNi-rGO Composite Electrodes for Use in Fuel Cells," *Catalysts*, vol. 14, no. 8, Aug. 2024, doi: 10.3390/catal14080551.

[22] E. Fumoto *et al.*, "Determination of carbonyl functional groups in lignin-derived fraction using infrared spectroscopy," *Fuel*, vol. 318, Jun. 2022, doi: 10.1016/j.fuel.2022.123530.

[26] K. S. Kim and S. J. Park, "Influence of multi-walled carbon nanotubes on the electrochemical performance of graphene nanocomposites for supercapacitor electrodes," *Electrochim Acta*, vol. 56, no. 3, 2011, doi: 10.1016/j.electacta.2010.10.043.

[32] J. L. E. Campos *et al.*, "Applications of Raman spectroscopy in graphene-related materials and the development of parameterized PCA for large-scale data analysis," *Journal of Raman Spectroscopy*, vol. 49, no. 1, 2018, doi: 10.1002/jrs.5225.
